# The venom-gland transcriptome of the eastern coral snake (*Micrurus fulvius*) reveals high venom complexity in the intragenomic evolution of venoms

**DOI:** 10.1186/1471-2164-14-531

**Published:** 2013-08-02

**Authors:** Mark J Margres, Karalyn Aronow, Jacob Loyacano, Darin R Rokyta

**Affiliations:** 1 Department of Biological Science, Florida State University, Tallahassee, FL 32306-4295, USA

## Abstract

**Background:**

Snake venom is shaped by the ecology and evolution of venomous species, and signals of positive selection in toxins have been consistently documented, reflecting the role of venoms as an ecologically critical phenotype. New World coral snakes (Elapidae) are represented by three genera and over 120 species and subspecies that are capable of causing significant human morbidity and mortality, yet coral-snake venom composition is poorly understood in comparison to that of Old World elapids. High-throughput sequencing is capable of identifying thousands of loci, while providing characterizations of expression patterns and the molecular evolutionary forces acting within the venom gland.

**Results:**

We describe the *de novo* assembly and analysis of the venom-gland transcriptome of the eastern coral snake (*Micrurus fulvius*). We identified 1,950 nontoxin transcripts and 116 toxin transcripts. These transcripts accounted for 57.1% of the total reads, with toxins accounting for 45.8% of the total reads. Phospholipases A_2_ and three-finger toxins dominated expression, accounting for 86.0% of the toxin reads. A total of 15 toxin families were identified, revealing venom complexity previously unknown from New World coral snakes. Toxins exhibited high levels of heterozygosity relative to nontoxins, and overdominance may favor gene duplication leading to the fixation of advantageous alleles. Phospholipase A_2_ expression was uniformly distributed throughout the class while three-finger toxin expression was dominated by a handful of transcripts, and phylogenetic analyses indicate that toxin divergence may have occurred following speciation. Positive selection was detected in three of the four most diverse toxin classes, suggesting that venom diversification is driven by recurrent directional selection.

**Conclusions:**

We describe the most complete characterization of an elapid venom gland to date. Toxin gene duplication may be driven by heterozygote advantage, as the frequency of polymorphic toxin loci was significantly higher than that of nontoxins. Diversification among toxins appeared to follow speciation reflecting species-specific adaptation, and this divergence may be directly related to dietary shifts and is suggestive of a coevolutionary arms race.

## Background

Snake venom is a cocktail of biologically active proteins with multifarious pharmacological effects representing the inverse of the physiological processes that maintain prey homeostasis [1]. Venom defines the ecology, life history, and evolution of venomous species due to its involvement in prey capture, digestion, and defense [1]. Positive selection has been repeatedly detected in toxin genes and reflects the significant contribution of venoms to fitness [2-4]. These molecular signals of adaptive evolution coupled with compositional variation suggest that toxin diversification is an adaptation to diet and may reflect a predator-prey arms race [5]. Snake venom components exert selective pressures on both prey [6,7] and predators [1], and potentially offer a unique, dual perspective on predator-prey coevolution.

New World coral snakes (Elapidae) consist of three genera (*Leptomicrurus, Micrurus,* and *Micruroides*) and over 120 species and subspecies, inhabiting a diverse array of habitats from the southern United States to central Argentina [8]. The eastern coral snake (*Micrurus fulvius*) is native to the forests of the southeastern United States and is mainly ophiophagous, preying upon smaller snakes and other squamates [9]. Bites from *Micrurus* species can be lethal [10] due to the pre- and postsynaptic effects of the neurotoxic venom components that dominate coral snake venoms (LD_50_ = 7–76 *μ*g venom/18–22 g mouse [11]), and polyvalent antivenom has been shown to be ineffective at treating bites from all *Micrurus* species [11,12] and is currently unavailable in the United States [10]. A full characterization of all venom components may allow the design of more effective polyvalent antivenom [13,14], but coral-snake venom composition is poorly understood in comparison to that of Old World elapids [15], mainly due to the difficulty of procuring sufficient venom quantities during milking for standard proteomic techniques [13]. High-throughput sequencing approaches are capable of identifying thousands of loci, enabling a detailed examination of the evolutionary forces shaping venom composition at the molecular level. We describe the first high-throughput transcriptomic characterization of an elapid venom gland to date. We sequenced the venom-gland transcriptome of *M. fulvius* with Illumina technology using the paired-end approach of Rokyta et al. [16], and used the generated sequence data to examine the relationship between toxin heterozygosity and gene duplication events and uncover distinct expression patterns in highly expressed and extremely diverse toxin gene families.

## Results and discussion

### High venom complexity revealed by means of sequencing

**Figure 1:**
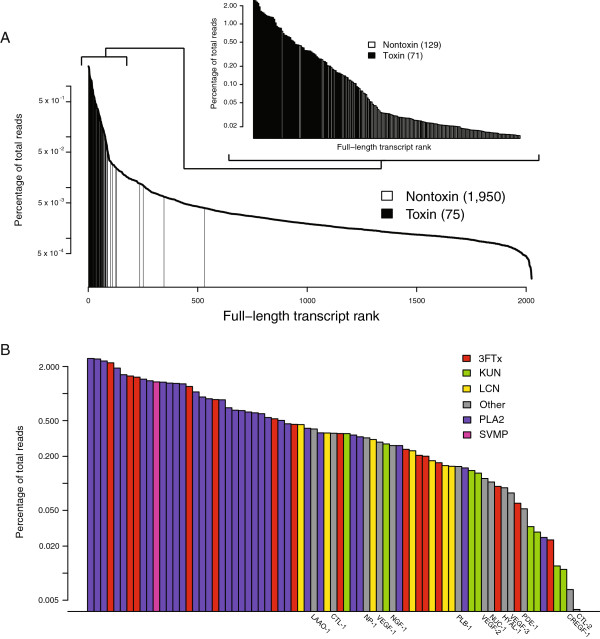
**The venom-gland transcriptome of*Micrurus fulvius* was extremely biased towards toxin production.** The venom-gland transcriptome of *M. fulvius* was dominated by toxin transcripts and, in particular, phospholipases A_2_ (PLA_2_s). **(A)** A total of 1,950 nontoxin-encoding and 116 toxin-encoding transcripts were identified. Toxins were grouped into 75 clusters based on <1% nucleotide divergence. The inset shows a magnification of the top 200 transcripts, the vast majority of which code for toxins. **(B)** Expression levels of individual toxin clusters, color coded by toxin class. The 75 toxin clusters represent 15 distinct toxin classes. Three-finger toxin and PLA_2_ transcripts dominated toxin expression levels, accounting for nearly 86% of all toxin reads. Toxin-class abbreviations are as follows: 3FTx: three-finger toxin; CTL: C-type lectin; CREGF: cysteine-rich with EGF-like domain; HYAL: hyaluronidase; KUN: Kunitz-type protease inhibitor; LAAO: L amino-acid oxidase; LCN: long-chain neurotoxin; NGF: nerve growth factor; NP: natriuretic peptide; NUC: nucleotidase; PDE: phosphodiesterase; PLA_2_: phospholipase A_2_; PLB: phospholipase B; SVMP: snake venom metalloproteinase; VEGF: vascular endothelial growth factor.

Our high-throughput transcriptomic analysis revealed high venom complexity in *M. fulvius*, comparable to the diversity of toxin components recently identified in the venom-gland transcriptome of the eastern diamondback rattlesnake (*Crotalus adamanteus*: Viperidae) [16]. We generated 79,573,048 pairs of 100 nucleotide raw reads and merged 61,609,456 pairs on the basis of overlap at their 3’ ends, following the approach of Rokyta et al. [16] and Rodrigue et al. [17]. These merged reads had an average length of 134 nucleotides with average phred scores of 46. The iterative assembly process described by Rokyta et al. [16] coupled with a reference-based assembly using nontoxin transcripts previously described in the venom-gland transcriptome of *C. adamanteus* resulted in the identification of 1,950 unique, full-length nontoxin coding sequences and 116 unique, full-length toxin coding transcripts (Figure 1A).

Transcript abundances were estimated by mapping 10 million reads to unique, full-length sequences with a minimum match percentage of 95% as described in Rokyta et al. [16] for *C. adamanteus*, against whose results we will be making comparisons. Toxin transcripts were grouped into clusters based on <1% nucleotide divergence for abundance estimates. This enabled us to account for allelic variation, recent duplication events, and possible sequencing errors while also allowing reads to be mapped to a unique sequence rather than to multiple sequences, resulting in a more accurate estimation of abundance levels. Our estimates of abundance reflect mRNA levels and not necessarily protein abundances; the relationship between mRNA and protein levels is not always straightforward [18], and the construction of a complete genotype-phenotype map requires proteomic analyses (e.g., mass spectrometry).

**Figure 2:**
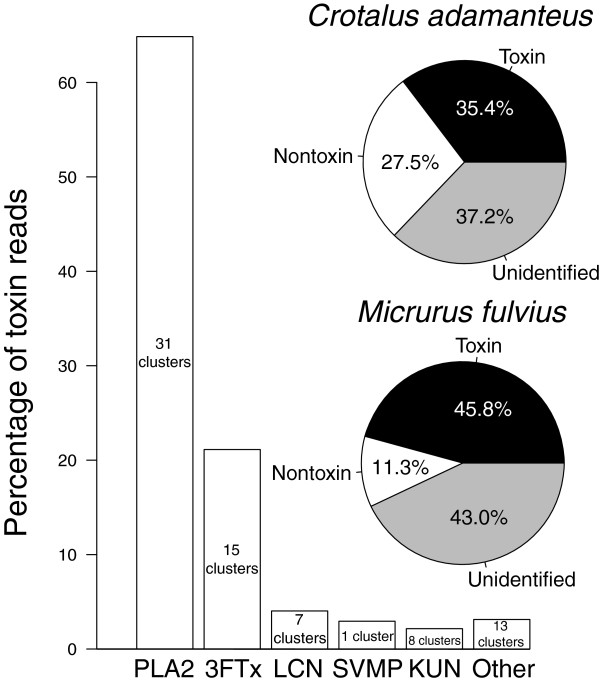
**The venom-gland transcriptome of *Micrurus fulvius * was dominated by phospholipases A**_**2**_** and three-finger toxins.** Toxin gene expression was dominated by phospholipases A_2_ (PLA_2_s) and three-finger toxins (3FTxs). Full-length transcripts accounted for 57.1% of the total reads; toxin sequences accounted for 45.8% of the total reads. PLA_2_s and 3FTxs represent both the most abundant and most diverse toxin classes identified; 31 PLA_2_ clusters accounted for 64.9% of the toxin reads, and 15 3FTx clusters accounted for 21.1% of the toxin reads. Toxin sequences accounted for 10.4% more of the total reads in *M. fulvius* than in *C. adamanteus*, while nontoxins in *C. adamanteus* accounted for more than twice the total read percentage than in *M. fulvius*. The venom of *M. fulvius* was dominated by small neurotoxic components while the venom of *C. adamanteus* was dominated by larger hemorrhage-inducing proteins, suggesting that the transcriptional effort expended on toxins versus nontoxins may differ between venoms dominated by high-molecular weight components and venoms dominated by smaller proteins, with small-component venoms expressing toxins at much higher levels relative to nontoxin production.

The 1,950 nontoxin transcripts accounted for 11.3% of the reads. The 116 full-length toxin transcripts were grouped into 75 clusters (Figure 1B, Table 1) and accounted for 45.8% of the reads. In total, we accounted for 57.1% of the reads (Figure 2), comparable to the amount identified for *C. adamanteus* using a similar approach [16]. While the overall percentage of reads mapping to identified transcripts was similar for *M. fulvius* and *C. adamanteus*, toxin expression levels were quite different (Figure 2). The toxin transcripts identified in *M. fulvius* accounted for nearly half of the total sequencing reads (45.8%) while the toxin transcripts in *C. adamanteus* accounted for approximately one-third (35.4%) of the total reads (Figure 2) [16]. The numbers and abundances of nontoxin coding sequences were much lower in *M. fulvius* than in *C. adamanteus*, despite an increase in assembly effort (e.g., the addition of the reference-based assembly), as 1,950 nontoxin transcripts accounted for 11.3% of the total reads in *M. fulvius* while 2,879 nontoxin transcripts accounted for 27.5% of the total reads in *C. adamanteus* (Figure 2). The venom-gland transcriptome of *C. adamanteus* was characterized by large, hemorrhage-inducing toxins such as snake venom metalloproteinases (SVMPs), proteins that presumably require extensive downstream processing by nontoxin machinery prior to becoming mature, active toxins [16]. The vast majority of highly expressed nontoxin sequences identified in the transcriptome of *C. adamanteus* were involved with proteostasis (e.g., protein folding, degradation, and transport) [16], and the reduction in the expression levels of nontoxin transcripts in *M. fulvius* could potentially reflect a difference in the maintenance, production, and folding requirements of the venom components of each species. The venom of *M. fulvius* was dominated by three-finger toxins (3FTxs) and phospholipases A_2_ (PLA_2_s), relatively short toxins that may not require the degree of downstream processing needed by larger toxins to become functional. This suggests that venoms dominated by smaller proteins differ in the transcriptional effort expended on toxins relative to nontoxins in comparison to venoms characterized by high-molecular weight components, with small-component venoms expressing toxins at much higher levels relative to nontoxin production. The largely proteinaceous composition of venom makes it metabolically costly to produce [19], and a reduction in the machinery necessary for the production of functional toxic proteins may confer an energetic benefit to species expressing smaller peptides and enzymes. Simple, smaller toxins have a reduced mutational target relative to larger proteins as a function of sequence length, potentially reducing the ability to evolve effective counterdefenses to resistance development in frequently envenomed prey [6,7] and predators [1] where more complex venoms would be advantageous. However, as our hypotheses are based on a comparison between a single representative of each family, sequencing additional members of Viperidae and Elapidae are needed to test whether these putative differences in transcriptional effort are fixed or unique to *M. fulvius* and/or *C.adamanteus*.

**Table 1:** Expression levels of toxin clusters

**Rank**	**Cluster name**	**Cluster size**	**Length**	**% toxin reads**	**% total reads**	**GenBank accession numbers**
1	PLA_2_-2	11	643	5.366	2.455	a: GAEP01002032
						b: GAEP01002033
						c: GAEP01002034
						d: GAEP01002035
						e: GAEP01002036
						f: GAEP01002037
						g: GAEP01002038
						h: GAEP01002039
						i: GAEP01002040
						j: GAEP01002041
						k: GAEP01002042
2	PLA_2_-5	2	826	5.294	2.422	a: GAEP01002053
						b: GAEP01002054
3	PLA_2_-16	1	815	5.036	2.304	GAEP01002014
4	3FTx-2	6	616	4.803	2.197	a: GAEP01001961
						b: GAEP01001962
						c: GAEP01001963
						d: GAEP01001964
						e: GAEP01001965
						f: GAEP01001966
5	PLA_2_-1	4	809	4.195	1.919	a: GAEP01002018
						b: GAEP01002019
						c: GAEP01002020
						d: GAEP01002021
6	PLA_2_-17	1	681	3.541	1.620	GAEP01002015
7	3FTx-1	4	394	3.424	1.567	a: GAEP01001957
						b: GAEP01001958
						c: GAEP01001959
						d: GAEP01001960
8	3FTx-3	2	532	3.327	1.522	a: GAEP01001967
						b: GAEP01001968
9	PLA_2_-11	1	1,534	3.162	1.447	GAEP01002009
10	PLA_2_-12	1	776	3.031	1.387	GAEP01002010
11	SVMP-1	1	2,323	2.947	1.348	GAEP01002063
12	PLA_2_-4	6	702	2.924	1.338	a: GAEP01002047
						b: GAEP01002048
						c: GAEP01002049
						d: GAEP01002050
						e: GAEP01002051
						f: GAEP01002052
13	PLA_2_-26	1	638	2.856	1.307	GAEP01002028
14	PLA_2_-24	1	760	2.835	1.297	GAEP01002026
15	PLA_2_-14	1	1,074	2.801	1.281	GAEP01002012
16	3FTx-4	2	568	2.617	1.197	a: GAEP01001969
						b: GAEP01001970
17	PLA_2_-3	2	874	2.278	1.042	a: GAEP01002045
						b: GAEP01002046
18	PLA_2_-8	1	1,846	2.001	0.915	GAEP01002060
19	PLA_2_-29	1	487	1.913	0.875	GAEP01002031
20	3FTx-6	1	382	1.873	0.857	GAEP01001973
21	PLA_2_-6	3	681	1.860	0.851	a: GAEP01002055
						b: GAEP01002056
						c: GAEP01002057
22	PLA_2_-20	1	825	1.518	0.695	GAEP01002022
23	PLA_2_-23	1	876	1.425	0.652	GAEP01002025
24	PLA_2_-7	2	782	1.411	0.645	a: GAEP01002058
						b: GAEP01002059
25	PLA_2_-27	1	550	1.361	0.623	GAEP01002029
26	PLA_2_-28	1	565	1.333	0.610	GAEP01002030
27	PLA_2_-13	1	754	1.302	0.596	GAEP01002011
28	PLA_2_-30	1	730	1.182	0.541	GAEP01002043
29	3FTx-7	1	603	1.145	0.524	GAEP01001974
30	PLA_2_-9	1	631	1.097	0.502	GAEP01002061
31	PLA_2_-22	1	666	1.006	0.460	GAEP01002024
32	3FTx-9	1	593	0.992	0.454	GAEP01001976
33	LCN-4	1	471	0.985	0.451	GAEP01001999
34	PLA_2_-25	1	515	0.897	0.410	GAEP01002027
35	LAAO-1	3	2,359	0.880	0.403	a: GAEP01001991
						b: GAEP01001992
						c: GAEP01001993
36	PLA_2_-19	1	985	0.798	0.365	GAEP01002017
37	LCN-1	3	613	0.795	0.364	a: GAEP01001994
						b: GAEP01001995
						c: GAEP01001996
38	CTL-1	2	939	0.790	0.361	a: GAEP01001978
						b: GAEP01001979
39	3FTx-11	1	496	0.784	0.359	GAEP01001952
40	KUN-2	1	551	0.782	0.358	GAEP01001984
41	PLA_2_-15	1	620	0.754	0.345	GAEP01002013
42	PLA_2_-31	1	506	0.720	0.330	GAEP01002044
43	NP-1	1	642	0.701	0.321	GAEP01002005
44	LCN-3	1	591	0.673	0.309	GAEP01001998
45	VEGF-1	1	4,642	0.629	0.288	GAEP01002064
46	KUN-1	2	421	0.600	0.274	a: GAEP01001982
						b: GAEP01001983
47	NGF-1	2	1,014	0.576	0.264	a: GAEP01002003
						b: GAEP01002004
48	PLA_2_-18	1	651	0.576	0.263	GAEP01002016
49	3FTx-14	1	377	0.522	0.239	GAEP01001955
50	LCN-6	1	548	0.504	0.231	GAEP01002001
51	3FTx-12	1	605	0.448	0.205	GAEP01001953
52	3FTx-13	1	492	0.439	0.201	GAEP01001954
53	LCN-2	1	734	0.390	0.179	GAEP01001997
54	3FTx-10	1	417	0.371	0.170	GAEP01001951
55	LCN-5	1	487	0.345	0.158	GAEP01002000
56	LCN-7	1	371	0.339	0.155	GAEP01002002
57	PLB-1	1	1,790	0.337	0.154	GAEP01002062
58	PLA_2_-21	1	668	0.325	0.149	GAEP01002023
59	KUN-7	1	442	0.303	0.139	GAEP01001989
60	KUN-3	1	442	0.284	0.130	GAEP01001985
61	VEGF-2	1	1,260	0.248	0.113	GAEP01002065
62	NUC-1	1	2,297	0.226	0.103	GAEP01002006
63	3FTx-15	1	625	0.202	0.092	GAEP01001956
64	HYAL-1	1	1,448	0.195	0.089	GAEP01001981
65	VEGF-3	1	1,422	0.171	0.078	GAEP01002066
66	3FTx-8	1	707	0.131	0.060	GAEP01001975
67	PDE-1	1	3,003	0.114	0.052	GAEP01002007
68	KUN-8	1	571	0.072	0.033	GAEP01001990
69	KUN-5	1	400	0.063	0.029	GAEP01001987
70	PLA_2_-10	1	1,120	0.055	0.025	GAEP01002008
71	3FTx-5	2	1,680	0.051	0.023	a: GAEP01001971
						b: GAEP01001972
72	KUN-6	1	304	0.026	0.012	GAEP01001988
73	KUN-4	1	856	0.024	0.011	GAEP01001986
74	CREGF-1	1	1,573	0.014	0.007	GAEP01001977
75	CTL-2	1	1,020	0.009	0.004	GAEP01001980

### Toxin class expression patterns

We identified 116 unique, full-length toxin transcripts representing 15 toxin classes or families which clustered into 75 groups with <1% nucleotide divergence. Clusters could include alleles, recent duplicates, or assembly/sequencing errors. These clusters accounted for 45.8% of the total reads (Figure 2), and nearly all of the high-abundance transcripts coded for toxins (Figure 1A). Of the 75 toxin clusters identified, 71 were among the top 200 most highly expressed genes (Figure 1A), while only 63 of the 78 toxin clusters identified in *C. adamanteus* were among the top 200 most highly expressed genes [16]. The expression patterns of the venom gland appear to be biased towards toxin production in *C. adamanteus*, but reach a more extreme level of specialization in *M. fulvius*. Although sequencing a single specimen may not accurately reflect gene expression for all individuals of *M. fulvius*, our analyses provide a reference transcriptome for future work with the species. We used the number of reads mapping to a specific transcript as an estimate of its abundance as per Rokyta et al. [16]. Toxin transcripts were named by the toxin-class abbreviation followed by the cluster number and a letter if multiple transcripts were present within the cluster.

#### Phospholipases A_2_

We identified 54 unique sequences of PLA_2_s that grouped into 31 clusters, which accounted for 64.9% of toxin reads and 29.7% of the total reads (Figure 2). PLA_2_s were the most abundant and diverse toxin class in the *M. fulvius* venom-gland transcriptome. PLA_2_s are esterolytic en- zymes and are among the most toxic components of snake venoms, causing pre- and postsynaptic neurotoxicity, myotoxicity, cardiotoxicity, hemolytic activity, anticoagulant activity, and hypotensive activity among other effects [20]. Conversely, SVMPs were the most abundant toxin gene family in *C. adamanteus*, with 16 clusters representing 39 sequences accounting for 24.4% of the toxin reads and 8.6% of the total reads [16]. PLA_2_s accounted for more than twice the percentage of toxin reads and over three times the percentage of total reads than the most abundant class in *C. adamanteus*, demonstrating the extreme dominance of gene expression by the PLA_2_ gene family in *M. fulvius*.

#### Three-finger toxins

We identified 26 full-length transcripts and 15 clusters of 3FTxs, which accounted for 21.1% of toxin reads and 9.7% of the total reads (Figure 2). 3FTxs were the second most abundant and diverse toxin class, and with PLA_2_s accounted for 86.0% of the total toxin reads. 3FTxs are short (60–71 amino-acid residues), nonenzymatic proteins characterized by three *β*-stranded loops extending from a center protein core [21]. These proteins are common components of elapid snake venoms and exhibit postsynaptic neurotoxicity by inhibiting the binding of acetylcholine to the muscle nicotinic acetylcholine receptors, causing a blockage of neuromuscular transmission [21]. The venom-gland transcriptome of *M. altirostris* was dominated by 3FTxs, as this toxin family accounted for approximately half of the identified expressed sequence tags [22]. In *M. fulvius*, 3FTxs only accounted for 9.7% of the total reads, reflecting vast differences in expression patterns within the genus.

#### Long-chain neurotoxins

Long-chain neurotoxins (LCNs) were the third most abundant and fourth most diverse toxin class. We identified nine sequences, which grouped into seven clusters and accounted for 4.0% of toxin reads and 1.8% of the total reads (Figure 2). LCNs are similar to the short-chain 3FTxs described above but contain an additional 6–12 amino acid residues and an additional disulfide bond [21]. While both antagonize muscle nicotinic acetylcholine receptors, differences in the functional sites of these toxins correlate to differences in the specificity of their targets [21]. LCNs were much more similar to one another than 3FTxs, being 5.3% divergent at the nucleotide level and 12.8% divergent at the amino acid level whereas divergence among members of the 3FTx class was extremely high, with a paucity of conserved nucleotides and amino acids across cluster members.

#### Snake venom metalloproteinases

SVMPs were the fourth most abundant toxin class, represented by a single transcript which accounted for 2.9% of the toxin reads and 1.3% of the total reads (Figure 2). SVMPs display extensive local and systemic hemorrhagic activity associated with envenomation in viperids, but the role of these enzymes following *Micrurus* envenomation is uncertain. These enzymes can be subdivided into classes based on differences in domain structure, and the transcript identified in the venom-gland transcriptome of *M. fulvius* is a member of the class SVMP-III, characterized by disintegrin-like, cysteine-rich, and metalloproteinase domains [23]. Larger molecular weight toxins, such as SVMPs, are often absent from elapid venoms and are more common components in the venoms of viperids [16,23]. High metalloproteinase expression has been shown to be incompatible with high toxicity in rattlesnakes, being described by Mackessy as a tradeoff between neurotoxicity and hemorrhagic activity [24]. High-molecular weight toxins such as SVMPs are the major causes of hemorrhage and necrosis commonly associated with viperid envenomations, and presumably aid in digestion and are most effective when localized at the site of the bite. Conversely, toxins with systemic effects (e.g., toxicity) such as 3FTxs are more effective when spread through the envenomated organism is rapid, resulting in prey immobilization. This dichotomy in pharmocological effects appears to hold in elapids as well, demonstrating the antagonistic effects of these components. Yet in comparison to other transcriptomic work with *Micrurus* species, SVMP expression levels are nearly five-times higher in *M. fulvius* than in congeneric individuals [22], suggesting a more active functional role for this toxin in *M. fulvius*. This hypothesis awaits proteomic verification and testing via comparative functional assays to quantify the activity of SVMPs in *M. fulvius* relative to congeners.

#### Kunitz-type inhibitors

Kunitz-type inhibitors (KUNs) were the fifth most abundant and third most diverse toxin class present in the *M. fulvius* transcriptome, with nine transcripts and eight clusters accounting for 2.2% of the toxin reads and 1.0% of the total reads (Figure 2). KUNs are characterized by possessing a compact tertiary fold and three disulfide bonds, functioning as both inhibitors of serine proteases and as neurotoxins by inhibiting calcium and potassium ion channels [25]. KUN-4 was a very divergent transcript, being 504 nucleotides longer than the next longest transcript. It shared 92.0% identity with a putative KUN known from the Australian elapid *Austrelaps labialis*[26] and 85.0% identity with a KUN identified in the venom-gland transcriptome of *C. adamanteus*[16]. This transcript is considered putative until a comprehensive functional characterization is completed. The removal of this extremely divergent transcript resulted in a maximum pair-wise nucleotide divergence of 31.0% within KUNs and a maximum pair-wise amino acid divergence of 68.0%, reflecting the diversity of this toxin class, especially in comparison to LCNs.

#### Low-abundance toxins

PLA_2_s, 3FTxs, LCNs, SVMPs, and KUNs accounted for 95.1% of the reads mapping to toxin sequences (Figure 1B), 82.7% of the toxin clusters, and 85.3% of the unique toxin transcripts. The remaining low-abundance toxins fell into ten different classes, are listed under “Other” in Figure 1B and Figure 2, and are described in Table 2.

**Table 2:** Low abundance toxin classes

**Toxin class**	**Clusters**	**Sequences**	**% toxin reads**	**% total reads**	**Putative function**
CTL	2	3	0.80	0.365	Disruption of hemostasis [27]
CREGF	1	1	0.01	0.007	Substrate recognition and specificity [28]
HYAL	1	1	0.20	0.089	Degradation of extracellular matrix and
					connective tissue; spreading factor [29]
LAAO	1	3	0.88	0.403	Edema, platelet aggregation, and apoptosis
					due to the liberation of peroxides [30]
NP	1	1	0.70	0.321	Hypotension [3]
NGF	1	2	0.58	0.264	Induction of apoptosis in neuronal cells [31]
NUC	1	1	0.23	0.103	Liberation of toxic nucleosides [32]
PDE	1	1	0.11	0.052	Hydrolysis of phosphodiester bonds; hypotension [32]
PLB	1	1	0.34	0.154	Unknown; potentially hemolytic and/or cytotoxic [2,33]
VEGF	3	3	1.05	0.479	Hypotension and augmentation of vascular permeability [34]

Abbreviations. *CTL* C-type lectin, *CREGF* Cysteine-rich with EGF-like domain, *HYAL* hyaluronidase, *LAAO* L amino-acid oxidase, *NP* natriuretic peptide, *NGF* nerve growth factor, *NUC* nucleotidase, *PDE* phosphodiesterase, *PLB* phospholipase B, *VEGF* vascular endothelial growth factor.

### Heterozygosity and gene duplication

Single nucleotide polymorphisms (SNPs) were identified in the coding regions of toxin clusters and nontoxin sequences using the SeqMan module from the DNAStar Lasergene software suite following a templated alignment in NGen. We analyzed 2,025 contigs and detected 98 SNPs in 78 transcripts (Table 3). Of these 78 transcripts, 69 coded for nontoxin proteins while the remaining nine SNP-containing transcripts were identified as toxins. Ten of the 69 nontoxin transcripts and two of the nine toxin sequences with variable sites contained multiple SNPs. A total of 87 SNPs were spread over 3.7% of nontoxin transcripts while 11 SNPs were found over 12.0% of toxin cluster sequences. Of the SNPs identified in nontoxin transcripts, 26.4% resulted in alterations to the amino-acid sequence, while 45.5% of the SNPs identified in toxin transcripts resulted in a nonsynonymous mutation. Overdominant selection favors polymorphism and increases genetic variability. Synonymous SNPs are often predicted to be invisible to the sieve of selection, and elevated nonsynonymous rates at polymorphic toxin loci suggest that the diversity of toxin genes can at least, in part, be explained by overdominant selection. Our SNP calling approach only identified polymorphic sites as SNPs if the nucleotide occurred with a frequency ranging from 40–60%. Therefore, sequences possessing SNPs were considered putative heterozygous loci, and we compared the frequency of heterozygous loci between classes to a *χ*^2^ distribution with one degree of freedom. Toxin and nontoxin sequences accounted for 5.6% and 94.4% of the total annotated transcripts, respectively. The frequency of heterozygous toxin loci was significantly greater than the frequency of heterozygous nontoxin sequences ( *χ*^2^ = 6.383,*df* = 1,*p* = 0.0115). Toxin transcripts also possessed a much higher SNP density than nontoxins. SNP density was calculated as the number of SNPs per 1,000 bases (kb) in the coding regions of toxin and nontoxin transcripts containing SNPs [35]. Nontoxin transcripts had a SNP density of 0.96 SNPs/kb while toxins had a SNP density of 1.93 SNPs/kb.

**Table 3:** Putative SNPs in 69 of the 1,950 nontoxin and nine of the 75 toxin sequences

**Transcript**	**Type**	**Coverage**	**Position**	**SNP%**	**Amino acid**	
40S ribosomal protein S14	Nontoxin	762	292	52.0	D	
40S ribosomal protein S18	Nontoxin	284	424	47.2	N	
40S ribosomal protein S23	Nontoxin	510	97	51.2	K	
6-phosphogluconate dehydrogenase	Nontoxin	33	624	54.5	V	
60S ribosomal protein L29	Nontoxin	411	45	51.3	K	
60S ribosomal protein L32	Nontoxin	525	332	47.8	K	
Actin-related protein 2/3 complex subunit 3	Nontoxin	30	255	50.0	R	
Alanine aminotransferase 2-like protein	Nontoxin	23	241	43.5	T	
Alpha globin	Nontoxin	26	435	50.0	F →L	
Aminopeptidase N	Nontoxin	32	2,592	46.9	R →C	
Annexin A2	Nontoxin	66	302	42.4	T →A	
Annexin A4	Nontoxin	286	248	44.4	A	
Annexin A5	Nontoxin	99	236	50.5	S	
ATP synthase H + transporting mitochondrial F0	Nontoxin	109	178	45.9	L	
Bifunctional heparan sulfate N-deacetylase/	Nontoxin	20	2,445	45.0	M →T	
N-sulfotransferase 2-like protein						
C4orf34	Nontoxin	1,021	175	48.5	A	
Caprin-1	Nontoxin	46	1,327	45.7	S →P	
Cell cycle progression protein 1	Nontoxin	22	708	45.5	G	
		52	2,286	42.3	K →E	
Cellular nucleic acid-binding protein	Nontoxin	83	156	56.6	T	
Chloride intracellular channel protein 1	Nontoxin	112	447	58.0	D	
Clathrin interactor 1	Nontoxin	36	1,731	50.0	P	
Cyclic AMP-responsive element-binding protein 3	Nontoxin	25	482	44.0	D	
Cytochrome c oxidase subunit 4	Nontoxin	76	227	47.4	E →A	
Dynactin subunit 6 1	Nontoxin	35	424	51.4	I	
Ectonucleoside triphosphate diphosphohydrolase 7	Nontoxin	27	901	48.1	K →E	
Endoplasmic reticulum oxidoreductin 1-Lbeta	Nontoxin	260	1,247	54.6	G	
EPS8	Nontoxin	49	2,112	49.0	Q →H	
Eukaryotic initiation factor 4A-1	Nontoxin	29	409	44.8	H	
		24	496	41.7	R	
		32	559	53.1	D	
Eukaryotic translation elongation factor 1 gamma	Nontoxin	313	908	50.5	I	
Eukaryotic translation initiation	Nontoxin	43	73	41.9	I	
		40	1,067	47.5	H	
Eukaryotic translation initiation factor 2 subunit	Nontoxin	37	1,172	51.4	L	
Eukaryotic translation initiation factor 3	Nontoxin	42	3,200	52.4	G	
Eukaryotic translation initiation factor 5A-1	Nontoxin	71	77	45.1	D	
		75	86	45.3	T	
		98	143	48.0	N	
		139	384	56.1	R	
		108	447	52.8	S	
		72	486	54.2	T	
Fructose-1,6-bisphosphatase 1	Nontoxin	54	1,078	44.4	R	
GABA	Nontoxin	31	144	45.2	R	
		42	159	54.8	D	
Guanine nucleotide-binding protein	Nontoxin	30	569	46.7	A →T	
Guanine nucleotide-binding protein subunit beta-2	Nontoxin	541	149	51.2	T	
Hydroxyacylglutathione hydrolase	Nontoxin	21	190	52.4	W →R	
		23	709	47.8	S →P	
Integrin-linked protein kinase	Nontoxin	29	1,205	55.2	A	
Interferon-related developmental regulator 2	Nontoxin	22	709	45.5	A →T	
		23	709	47.8	S →P	
Integrin-linked protein kinase	Nontoxin	29	1,205	55.2	A	
Interferon-related developmental regulator 2	Nontoxin	22	709	45.5	A →T	
LAG1 longevity assurance protein 2	Nontoxin	41	896	53.7	T	
LAMTOR2	Nontoxin	81	468	53.1	L	
LIM domain and actin-binding protein	Nontoxin	28	1,516	50.0	V →A	
Lysine-specific histone demethylase 1B	Nontoxin	70	122	44.3	D →G	
Lysosomal cobalamin transporter	Nontoxin	27	184	48.1	A	
Malate dehydrogenase	Nontoxin	68	1,059	57.4	L	
Mannose-1-phosphate guanyltransferase beta	Nontoxin	32	698	53.1	P →S	
Microspherule protein 1	Nontoxin	24	665	50.0	V →A	
Minor histocompatibility antigen H13 1	Nontoxin	452	1,164	53.5	P →L	
MTP4	Nontoxin	1,664	503	59.3	D	
		1,480	1,621	49.8	P →S	
NADH dehydrogenase	Nontoxin	40	213	40.0	I →V	
NADPH-cytochrome P450 oxidoreductase	Nontoxin	28	286	53.6	T	
Nuclease-sensitive element-binding protein 1	Nontoxin	91	675	56.0	R	
Polyubiquitin-C isoform 2	Nontoxin	892	350	57.4	G	
		637	560	52.3	F	
		593	635	51.8	K	
		774	917	52.8	A	
		697	950	49.6	S	
Proteasome	Nontoxin	83	430	59.0	F	
Proteasome subunit alpha type-6	Nontoxin	48	139	52.1	H	
Protein disulfide-isomerase A3	Nontoxin	146	196	50.0	C	
Sarcolemmal membrane-associated protein-3	Nontoxin	21	1,534	42.9	Q	
SEC31	Nontoxin	110	2,866	46.4	P	
Serine/threonine-protein phosphatase	Nontoxin	22	1,144	40.9	T →S	
		22	1,337	45.5	S	
Sialin	Nontoxin	23	321	43.5	P	
Sodium/glucose cotransporter 4	Nontoxin	26	579	57.7	S →C	
		26	580	57.7	S →T	
Stress-induced-phosphoprotein 1	Nontoxin	22	455	50.0	L	
		21	716	52.4	K	
Transmembrane emp24 domain-containing	Nontoxin	347	595	46.1	Y	
Tubulin beta-5 chain	Nontoxin	27	1,340	44.4	A	
V-type proton ATPase catalytic subunit A	Nontoxin	63	1,344	55.6	I	
Y box binding protein 1	Nontoxin	91	811	59.3	R	
YTH domain family protein 1	Nontoxin	25	1,347	44.0	P	
C-type lectin 1a	Toxin	7,320	360	55.1	G	
C-type lectin 2	Toxin	130	598	48.5	N	
Cysteine-rich with EGF-like domain 1	Toxin	48	257	50.0	K	
		82	949	45.1	T	
Kunitz inhibitor 1a	Toxin	12,402	280	42.5	G →R	
L-amino acid oxidase 1a	Toxin	1,850	769	55.0	Y	
		3,094	1,158	41.2	H →Q	
Long chain neurotoxin 3	Toxin	7,081	402	40.1	V →I	
Phospholipase A_2_ 31	Toxin	20	62	40.0	S	
Phospholipase A_2_ 9	Toxin	68	44	50.0	N →I	
Venom nerve growth factor 1a	Toxin	3,689	316	49.3	P →S	

Sequences in multitranscript clusters appear to represent different allelic states of heterozygous loci, potentially reflecting how heterozygote advantage can favor gene duplication. If duplicate genes confer an advantage by providing more of a specific gene product, duplication events involving genes that are already highly expressed would be most beneficial [36]. While duplication events of highly expressed toxin transcripts within the snake venom gland have been rampant throughout the evolutionary history of venomous species (e.g., the formation of large gene families), this does not explain the increased heterozygosity detected in our analyses. Gene duplication may also produce a selective advantage when overdominance occurs. In this scenario, carrying different alleles at a single locus is beneficial and gene duplication can ultimately result in the fixation of multiple advantageous alleles. Heterozygosity occurred with a significantly higher frequency in toxin than in nontoxin sequences with nearly half of the polymorphic sites in toxins resulting in a nonsynonymous mutation, suggesting that heterozygote advantage may play a key role in driving gene duplication and allelic variation within toxin genes [37] (but see [38]).

### Expression biases and sequence divergence following speciation

**Figure 3:**
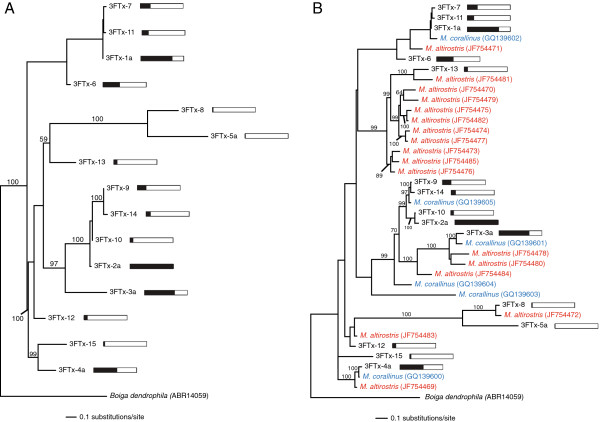
**The expression of three-finger toxins was extremely biased in*Micrurus fulvius*.** Expression of three-finger toxin (3FTx) sequences was dominated by several multitranscript cluster members, suggesting a significant role for recent gene duplication within the gene family. A homologous toxin sequence from the mangrove snake (*Boiga dendrophila*) was used as an outgroup to root the phylogeny. Bayesian posterior probability values exceeding 50% are shown. Adjacent bars indicate expression levels relative to the most highly expressed member of the class, with a completely filled bar indicating the most highly expressed transcript of the class. **(A)** A maximum-likelihood phylogeny of *M. fulvius* 3FTx clusters under the HKY+G model. 3FTx expression levels were dominated by a handful of sequences, all representatives of multitranscript clusters (as indicated by the letter following the cluster number), suggesting a relationship between gene duplication and expression. **(B)** A maximum- likelihood phylogeny of *M. fulvius* transcripts identified in this study as well as orthologous 3FTx transcripts from *M. corallinus* and *M. altirostris* under the HKY+G model. Tips are color-coded by species. The subclade containing nine *M. altirostris* transcripts is sister to a sub-clade containing a single *M. altirostris* sequence and 3FTx-13 from *M. fulvius*. 3FTx-13 is expressed at a relatively low level, suggesting that the divergence of toxins occurs following speciation, at least for this one 3FTx paralog.

The maximum-likelihood phylogeny for 15 unique *M. fulvius* 3FTx cluster members was estimated under the HKY+G model (Figure 3A). Expression levels were extremely biased toward a handful of sequences; four transcripts accounted for more than 67.0% of the toxin reads mapping to 3FTxs. These high-abundance transcripts (3FTx-1a, 2a, 3a, and 4a) are all members of multitranscript clusters, and duplication events and the number of alleles appear to be positively correlated with expression within the 3FTx toxin gene family. The maximum-likelihood phylogeny for 15 *M. fulvius* 3FTx clusters and 23 3FTx transcripts from *M. altirostris* and *M. corallinus* was estimated under the HKY+G model (Figure 3B). Two of the four most highly expressed *M. fulvius* 3FTx paralogs are in well-supported clades con- taining orthologs from both *M. altirostris* and *M. corallinus*. 3FTx-13, while accounting for approximately 2.0% of the 3FTx reads in *M. fulvius*, is in a well supported clade containing ten *M. altirostris* sequences indicating that orthologs to 3FTx-13 have undergone duplication events in *M. altirostris*. *M. altirostris* sequences were identified in a venom-gland transcriptome analysis where the toxin family accounted for 52.8% of the 867 expressed sequence tags generated [22], demonstrating that these transcripts are highly expressed. The apparent dominance of the 3FTx toxin family in *M. altirostris* by these transcripts, and the extremely low expression of 3FTx-13 in *M. fulvius* could reflect dietary differences between the species, as shifts in prey consumption have been known to be coupled with expression modifications that augment venom efficacy [4]. Genes that are no longer effective are lost or silenced while effective genes are highly expressed and diversify, suggesting functional divergence among toxins occurs following speciation events [4].

**Figure 4:**
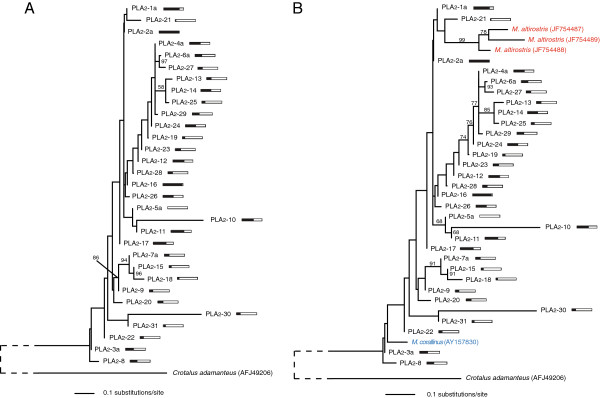
**The expression of phospholipases A**_**2**_** was uniform across transcripts in*Micrurus fulvius*.** The phospholipase A_2_ (PLA_2_) gene family was characterized by an even distribution of expression levels. A homologous toxin sequence from the eastern diamondback rattlesnake (*Crotalus adamanteus*) was used as an outgroup to root the phylogeny. The root branch was shortened for visual clarity and is indicated by the dashed line. Bayesian posterior probability values exceeding 50% are shown. Adjacent bars indicate expression levels relative to the most highly expressed member of the class, with a completely filled bar indicating the most highly expressed transcript of the class. **(A)** A maximum-likelihood phylogeny of *M. fulvius* PLA_2_ clusters under the SYM+G model. PLA_2_ expression levels were much more evenly distributed throughout the class in comparison to 3FTx transcript expression levels (Figure 4). **(B)** A maximum-likelihood phylogeny of *M. fulvius* transcripts identified in this study as well as orthologous PLA_2_ transcripts from *M. corallinus* and *M. altirostris* under the SYM+G model. Tips are color-coded by species. The sub-clade containing the *M. altirostris* transcripts again suggests divergence among toxins may occur following speciation events.

A maximum-likelihood phylogeny for 31 unique *M. fulvius* PLA_2_ clusters was estimated under the SYM+G model (Figure 4A). Expression levels for PLA_2_ transcripts were much more evenly distributed across the entire gene family in comparison to 3FTx sequences. The most highly expressed 3FTx transcript, 3FTx-2a, accounted for 22.7% of the total 3FTx reads while four transcripts accounted for approximately 67.0% of the 3FTx reads. In the PLA_2_ gene family, PLA_2_-2a was the most abundant toxin family member, accounting for 8.3% of the total PLA_2_ reads and the four most highly expressed transcripts accounted for only 30.8% of the reads mapping to PLA_2_s. Although these two families dominated toxin transcript expression levels in the *M. fulvius* venom gland, PLA_2_s did so through uniform expression while 3FTx expression patterns were tremendously biased. PLA_2_ transcripts also demonstrated less sequence divergence among clusters relative to 3FTx sequences. PLA_2_s are esterolytic enzymes that share a conserved three-dimensional structure [4,20], and the greater similarity between PLA_2_ transcripts may be a result of more stringent conformational constraints. The conservation of crucial structures in PLA_2_ enzymes ensures a functioning active site whereas the relatively short 3FTx peptides may be free of this limitation. Lynch [4] found that functionally critical sites were under strong purifying selection in PLA_2_s, with strong directional selection being restricted to surface residues due to their interactions with specific targets in prey, enabling prey-specific adaptation while ensuring the functionality of the enzyme. The maximum-likelihood phylogeny for 31 *M. fulvius* PLA_2_ clusters, three *M. altirostris* sequences, and a single *M. corallinus* sequence was estimated under the SYM+G model (Figure 4B). Functional divergence among PLA_2_s may also occur following speciation events [4], as all three *M. altirostris* sequences constitute a monophyletic clade and are sister to PLA_2_-21, a transcript that accounts for <1% of PLA_2_ reads in *M. fulvius* (although the pattern is not as strong as in 3FTxs as the PLA_2_-21/ *M. altirostris* clade is not well-supported).

Rampant gene duplication following the initial recruitment of toxin genes into the venom gland has led to the production of multigene toxin families [36], including the highly expressed 3FTx and PLA_2_ gene families present in the *M. fulvius* transcriptome. Gene duplication can be advantageous by increasing the production of a beneficial gene product or permanently fixing multiple advantageous alleles. Gene duplication can be followed by elevated rates of selection [39], enabling genes to acquire new functions or divide single functions among several genes [36]. Phylogenetic analyses of the 3FTx and PLA_2_ toxin classes (Figures 3 and 4) demonstrate the pervasiveness of gene duplication throughout the evolutionary history of these toxin families, and suggest that the divergence of toxin genes occurs following speciation. Zhang [36] stated that species-specific duplication events can result in species-specific gene function and, subsequently, adaptation. This adaptation can result in divergence and the development of unique phenotypes [36], which may be reflected in our phylogenetic analyses of multiple *Micrurus* species.

### Intragenomic evolution of venom genes

Positive selection in toxin genes has been repeatedly documented, reflecting the significance of venom to the fitness of venomous species [2-4]. Selection analyses have taken a somewhat unsystematic approach, analyzing all sequences available with little regard for the evolutionary histories of the taxa involved [4,40]. Here, we examine the intragenomic evolution of toxin classes within the *M. fulvius* genome. The codeml results for detecting the presence of positive selection are summarized in Table 4. The results from the M7 and M8 models are nearly identical to the results from the M1 and M2 models and are therefore not shown. We examined 3FTxs, KUNs, LCNs, and PLA_2_s for the presence of positive selection as these represent the four most diverse and four of the five most abundant toxin classes identified. While the fourth most highly expressed toxin class was SVMPs, this class was represented by a single transcript and thus could not be included in our analyses.

**Table 4:** Summary of codeml results

**Class**	***n*/Model**	**M1: Nearly neutral**	**−ln*L***	**M2: Positive selection**	**−ln*L***	**M0:*ω***	***λ***	***p-value***
3FTx	26	*p*: 0.27 0.73	1,600.40	*p*: 0.13 0.31 0.56	1,576.50	1.48	47.8	4.17 ×10^−11^*
	F81+G	*ω*: 0.11 1.00		*ω*: 0.00 1.00 3.79				
KUN	9	*p*: 0.32 0.68	965.08	*p*: 0.31 0.45 0.24	962.16	0.58	5.84	5.39 ×10^−2^
	GTR+G	*ω*: 0.02 1.00		*ω*: 0.03 1.00 2.74				
LCN	9	*p*: 0.86 0.14	315.85	*p*: 0.84 0.00 0.16	305.84	999.00	20.02	4.49 ×10^−5^*
	JC+I	*ω*: 1.00 1.00		*ω*: 0.00 1.00 999.00				
PLA_2_	52	*p*: 0.66 0.34	4,761.33	*p*: 0.49 0.28 0.23	4,359.71	3.32	443.24	0.00*
	SYM+G	*ω*: 0.05 1.00		*ω*: 0.04 1.00 11.68				

*dN/dS* ratios are represented by *ω*; *λ* represents our test statistic which is negative twice the difference in log likelihoods compared across models to a *χ*^2^ distribution with two degrees of freedom; and *p* corresponds to the proportion of codon sites falling into one of three rate classes which are purifying selection, neutral selection, and positive selection, respectively. The model selected by MrModelTest2.3 [50] for the estimation of the maximum-likelihood phylogeny is given and *n* corresponds to the number of sequences used in the analysis. Significant p-values are indicated by an asterisk.

The M0 model estimates a single *ω* value over all branches in the phylogeny and therefore is only capable of detecting evidence of selection when the majority of sites have experienced positive selection throughout their evolutionary histories [4]. Under the M0 model, we detected strong evidence of positive selection ( 1.48≤*ω*≤999.00) for the 3FTx, LCN, and PLA_2_ toxin families. Evidence of positive selection was not detected in the KUN toxin family ( *ω*=0.58), and the extraordinarily high *ω* calculated for the LCN family ( *ω*=999.00) was a result of all polymorphic sites resulting in nonsynonymous substitutions. We also used site specific models (M1, M2, M7, and M8) to detect rate variation among sites [2,4]. The 3FTx, LCN, and PLA_2_ toxin classes rejected the null or nearly neutral model (M1) in favor of the selection model (M2) ( 0.00≤*p*≤4.49×10^−5^), demonstrating the pervasiveness of positive selection experienced by highly expressed toxin transcripts within the *M. fulvius* genome. The percentage of sites experiencing selection ranged from 16–56% with 3.79≤*ω*≤999.00. The KUN toxin class did not reject the M1 model in favor of the selection model ( *p*=0.0539). The high *ω* value under the site specific models for the LCN class again reflects the absence of synonymous substitutions within the class. Elevated rates of evolution have previously been documented in toxin genes [2,4,41,42], and this accelerated evolution is most likely due to their direct involvement in fitness [2,4] and may be reflective of a coevolutionary arms race with specific prey taxa [6,7].

### Sequence accession numbers

The original, unmerged reads were submitted to the National Center for Biotechnology Information (NCBI) Sequence Read Archive under accession number SRA062772. The annotated sequences were submitted to the GenBank Transcriptome Shotgun Assembly database under accession numbers GAEP01000001–GAEP01001950 (nontoxins) and GAEP01001951–GAEP01002066 (toxins).

## Conclusions

We have described the most comprehensive transcriptomic characterization of an elapid venom gland to date, revealing venom complexity previously unknown from any New World coral snake [11,13,15,22,43-45]. Transcriptional effort expended on toxins relative to nontoxins may differ between venoms dominated by high-molecular weight components and venoms dominated by smaller proteins. This reduction in the machinery required for the production of functional toxins may confer a metabolic advantage to species expressing smaller peptides and enzymes, but may also reduce the capacity of species to evolve effective counterdefenses to resistant prey or predators. Toxin expression was dominated by PLA_2_s and 3FTxs, yet these two gene families greatly differed in expression patterns. Expression within the 3FTx family was extremely biased, being dominated by a handful of transcripts while PLA_2_ expression was much more uniform. SNP analysis revealed the frequency of heterozygous loci was significantly higher in toxins than in nontoxins with nearly half of the polymorphic sites in toxins resulting in a nonsynonymous substitution, suggesting overdominance may ultimately favor gene duplication and permanent fixation of advantageous alleles within the venom gland. We detected evidence of positive selection in three of the four most diverse and highly expressed toxin classes; sequence evolution or modifications of toxin expression patterns could increase the specificity of venoms for frequently envenomed prey items [5]. Diet has been proposed to be an important selective regime in determining venom composition within *Micrurus* species [46], and elevated rates of selection suggest a coevolutionary arms race [1].

## Methods

### Venom-gland transcriptome sequencing

We followed the approach described in Rokyta et al. [16] for the preparation and sequencing of the venom gland. We sequenced the venom-gland transcriptome of an adult female *M. fulvius* from Wakulla County, FL, with a snout-to-vent length of 620 mm and a total length of 685 mm. The snake was anesthetized by propofol injection (10 mg/kg), and venom was extracted by electrostimulation [47]. The animal was allowed to recover for four days for transcription to be maximized [48], at which point the snake was euthanized by sodium pentobarbitol injection and its venom glands removed [16]. This procedure was approved by the Florida State University Institutional Animal Care and Use Committee under protocol #0924. Sequencing and nonnormalized cDNA library preparation was performed by the HudsonAlpha Institute for Biotechnology Genomic Services Laboratory (http://www.hudsonalpha.org/gsl/). Total RNA was reduced to poly-A + RNA with oligo-dT beads. Two rounds of poly-A + selection were performed. The mRNA then underwent a mild heat fragmentation followed by random priming for first-strand synthesis. Standard second-strand synthesis was followed by library preparation with the double-stranded cDNA as input material. Sequencing was performed in a single lane on the Illumina HiSeq 2000 with 100-base-pair, paired-end reads.

### Transcriptome assembly and analysis

We followed the general approach described in Rokyta et al. [16] for the *de novo* assembly of the *M. fulvius* venom-gland transcriptome. Most of our read pairs overlapped at the 3’ end and these reads were subsequently merged to produce longer, composite reads [16,17]. Quality scores were updated accordingly and only these merged reads were used in the assembly process. The Extender program [16] was used as a *de novo* assembler of 1,000 random reads to eliminate extremely high-abundance transcripts. Full-length transcripts were identified with blastx searches and annotated. These sequences were then used as a template to filter a set of the unassembled, original merged reads using NGen 3.1 and a minimum match percentage of 98%. This *de novo* approach using the Extender program was repeated a second time as described above, using 1,000 of the filtered reads as the program continued to be productive at assembling full-length, unique transcripts. We next identified nontoxin transcripts by aligning 10 million of the unassembled reads to nontoxin transcripts previously identified in the venom-gland transcriptome of *C. adamanteus*[16] using the NGen assembler. We performed an initial alignment with a minimum match percentage of 93%. We then performed a second alignment with a reduced minimum match percentage of 90% to assemble more divergent transcripts. All other parameter values were consistent with our *de novo* assemblies in NGen. Coding regions with minimum ten-fold coverage were annotated, and all regions below this threshold were removed. These transcripts were combined with the Extender results and this unique set of sequences was then used as a template to filter a set of the merged reads using NGen 3.1 and a minimum match percentage of 98%. Next, 10 million filtered reads were used in a *de novo* assembly with NGen 3.1 with a minimum match percentage of 93%. Contigs with a minimum of 200 reads were identified with blastx searches, annotated, and duplicates removed, producing a unique set of identified transcripts. This process of filtering, NGen assembly, and annotating was performed an additional two times. Abundances were estimated by mapping 10 million merged reads to full-length sequences with a minimum match percentage of 95% using a reference-based assembly in NGen [16]. The percentage of reads mapping to an individual transcript was used to estimate abundance. Toxin transcripts were clustered based on <1% nucleotide divergence for abundance estimates.

### Detecting heterozygosity

Ten million merged reads were aligned to the 2,025 annotated transcripts in a reference-based assembly in NGen with a minimum match percentage of 95%. SNPs were identified by using the SeqMan module of the DNAStar Lasergene software suite. Toxin transcripts were clustered based on <1% nucleotide divergence as SNP detection provided an approach to identify allelic variation within toxin clusters. SNPs were only considered if they occurred in the coding sequence of full-length, annotated transcripts, had a SNP% ranging from 40–60%, and at least 20-fold coverage with maximum coverage bounded at 20,000-fold. These parameters are more stringent than previous SNP identification approaches [35] and provide a conservative estimation of variable sites in the coding regions of annotated transcripts. The frequency of toxin versus nontoxin heterozygous loci, relative to the number of transcripts belonging to each class identified in the transcriptome, was compared to a *χ*^2^ distribution with one degree of freedom.

### Phylogenetic analyses

Sequences of 3FTxs and PLA_2_s were independently aligned on the basis of the amino-acid sequence in the MegAlign module of the DNAStar Lasergene software suite with ClustalW [49]. Model selection was performed using the Akaike Information Criterion values with MrModelTest2.3 [50], and maximum-likelihood phylogenies were estimated using PAUP* version 4.0a126 [51] and the iterative search strategy described by Rokyta et al. [52]. Nodal support was determined in MrBayes v3.1.2 by the estimation of Bayesian posterior probabilities [53,54]. Markov Chain Monte Carlo searches were run for 10 million generations with four chains and a temperature parameter of 0.20. Samples were taken every 1,000 generations and the first million generations were discarded as burn-in. A related transcript from the mangrove snake (*Boiga dendrophila*: Colubridae) served as the outgroup to root each 3FTx phylogeny while a transcript from the eastern diamondback rattlesnake (*C. adamanteus*) served as the outgroup to root each PLA_2_ phylogeny. Orthologous transcripts of both toxin families from congeners were downloaded from the NCBI database and included in a second analysis following the method described above.

### Detecting selection

Transcripts from the four major toxin families (3FTx, KUN, LCN, and PLA_2_) were used in selection analyses. Sequences were independently aligned according to class on the basis of the amino-acid sequence in the MegAlign module of the DNAStar Lasergene software suite with ClustalW [49]. Gaps, stop codons, and signal peptides were excluded from all analyses, and only transcripts possessing signal peptides were included. Signal peptides mediate the targeting and transporting of the pre-protein and are cleaved prior to expression [55]. Their exclusion from our analyses ensured that only the mature amino-acid sequences of expressed toxins that are targets of selection were examined. Model selection was performed using the Akaike Information Criterion values with MrModelTest2.3 [50]. A maximum likelihood phylogeny was constructed using PAUP* version 4.0a126 [51] and the iterative search strategy previously described by Rokyta et al. [52].

A likelihood-ratio test for positive selection was conducted with codeml from the PAML version 4.4 package [56,57] with the maximum-likelihood phylogeny estimated as described above [2]. The null model, or the nearly neutral model (M1), allows for a class of sites to be evolving under neutral selection (*dN/dS*=1) while constraining the *dN/dS* for a second class to be <1. The alternative model, or the positive selection model (M2), incorporates an additional class that allows for a proportion of codon sites to be experiencing positive selection (*dN/dS* >1). To test for the presence of positive selection, negative twice the difference in log likelihoods were compared between models to a *χ*^2^ distribution with two degrees of freedom. A similar test was also performed to verify the results of the initial analysis, comparing models M7 (Beta) and M8 (Beta with positive selection) to a *χ*^2^ distribution with two degrees of freedom [58]. To estimate an overall *dN/dS*, the M0 model was used. This model averages the *dN/dS* across the entire phylogeny, producing a single ratio for all sites. While this model has been shown to have limited power at detecting positive selection [59], it provides a broader perspective and a more conservative estimate of selection within the *M. fulvius* genome than the site-specific models described above.

## Abbreviations

3FTx: Three-finger toxin; CTL: C-type lectin; CREGF: Cysteine-rich with EGF-like domain; HYAL: Hyaluronidase; kb: Kilobase; KUN: Kunitz-type protease inhibitor; LAAO: L amino-acid oxidase; LCN: Long-chain neurotoxin; NGF: Nerve growth factor; NP: Natriuretic peptide; NUC: Nucleotidase; PDE: Phosphodiesterase; PLA2: Phospholipase A2; PLB: Phospholipase B; RP-HPLC: Reverse-phase high-performance liquid chromatography; SNP: Single nucleotide polymorphism; SVMP: Snake venom metalloproteinase.

## Competing interests

The authors declare that they have no competing interests.

## Authors’ contributions

The project was conceived and planned by DRR. MJM, KA, JL, and DRR collected and analyzed the data. MJM wrote the manuscript. All authors read and approved the final manuscript.
